# Association of Allelic Variants of the Reelin Gene with Autistic Spectrum Disorder: A Systematic Review and Meta-Analysis of Candidate Gene Association Studies

**DOI:** 10.3390/ijerph17218010

**Published:** 2020-10-30

**Authors:** Ignacio Hernández-García, Antonio-Javier Chamorro, Hugo Guillermo Ternavasio-de la Vega, Cristina Carbonell, Miguel Marcos, José-Antonio Mirón-Canelo

**Affiliations:** 1 Department of Preventive Medicine and Public Health, Lozano Blesa University Clinical Hospital of Zaragoza, Calle San Juan Bosco 15, 50009 Zaragoza, Spain; 2 Department of Medicine, University of Salamanca, 37007 Salamanca, Spain; ajchamorro@gmail.com (A.-J.C.); guillermoternavasio@gmail.com (H.G.T.-d.l.V.); carbonell@usal.es (C.C.); mmarcos@usal.es (M.M.); 3 Department of Internal Medicine, University Hospital of Salamanca, 37007 Salamanca, Spain; 4 Institute of Biomedical Research of Salamanca (IBSAL), 37007 Salamanca, Spain; miroxx@usal.es; 5 Department of Epidemiology, University of Salamanca, 37007 Salamanca, Spain

**Keywords:** reelin, autistic spectrum disorder, polymorphism, genetics, meta-analysis

## Abstract

Autistic spectrum disorder (ASD) is a complex neurodevelopmental disability with a genetic basis, and several studies have suggested a potential role of the reelin gene (RELN) in ASD susceptibility. Accordingly, genetic association studies have explored this potential association, but the results have been controversial thus far. For this reason, we assessed the association of four genetic variants of RELN (the 5′UTR CGG triplet repeat and polymorphisms rs736707, rs362691, and rs2229864) with ASD by means of a systematic review and meta-analysis. We retrieved studies comparing the distribution of the above-mentioned genetic variants between ASD patients and healthy controls. A meta-analysis was conducted using a random effects model, and calculations of the odds ratios (ORs) and confidence intervals (CIs) were performed. A sensitivity analysis and tests to determine the heterogeneity of the results were also performed. Eleven previous studies fulfilled the inclusion criteria and analyzed the association of the above-mentioned genetic variants and ASD. We did not find any significant association between the allele or genotype frequencies of the analyzed polymorphisms and ASD, and large heterogeneity was found for the rs736707 polymorphism. Moreover, no significant differences were found between the 5′UTR triplet repeat and this disorder. In light of current evidence, no single genetic variant within this gene is clearly associated with the development of ASD, and ethnic differences may explain part of the observed heterogeneity. Larger studies among different ethnic groups are needed to establish the role of specific genetic variants within RELN in the etiology of this disorder.

## 1. Introduction

Autistic spectrum disorder (ASD) is a group of complex neurodevelopment disorders, including autism, pervasive developmental disorder not otherwise specified, Asperger syndrome, and other related conditions [1,2]. This disorder is characterized by impairments in social interactions and communication, with stereotypical patterns of behaviors and activities [3,4]. The prevalence of this disorder is at least approximately 1.5% in developed countries. Specifically, the prevalence is 18.5 per 1000 (one in 54) children aged eight years in the United States of America [5]. In other countries, such as the United Kingdom and Italy, the prevalence of ASD is 15.7 per 1000 [6] children aged 5–9 years and 11.5 per 1000 children aged 7–9 years [7], respectively. In 2010, a review of 23 studies found that the estimated prevalence of ASD across Asian countries/territories (Japan, China, Iran, Taiwan, Israel, and Indonesia) varied from 1.1 to 21.8 per 10,000 [8]. The prevalence of ASD is increasing in Asia. Recently, it was estimated that the prevalence of ASD in East Asia (Korea, India, and China) is 0.51%, 0.31% in South Asia (Nepal and Sri Lanka), and 0.35% in West Asia (Israel, Lebanon, Bangladesh, and Iran). In particular, ASD prevalence ranges between 0.06% in Iran and 2.64% in Korea [9]. Other authors have described an ASD prevalence of 785 per 100,000 children younger than five years in North Africa [10]. Thus, there are discrepancies in the prevalence rates across cultures, although differences in the prevalence of ASD obtained in such studies may also be justified, in part, by differences in the diagnostic criteria or epidemiological sampling methods used [9]. 

ASD affects all ethnic and socioeconomic groups. It is often associated with pronounced personal suffering and a heavy burden of care to families and society [11]. Moreover, children with ASD are substantially afflicted by ASD-related outcomes, including co-existing disorders [12] and bullying [13]. Moreover, one out of six children with ASD has several degrees of development disability, which may lead to intellectual disability [3,4,5]. 

The etiology of ASD is far from completely elucidated, but twin and family studies strongly support a genetic component [3,14,15,16]. Some single nucleotide polymorphisms (SNPs), such as rs10099100 on chromosome 8 and rs1000177 on chromosome 20, have been associated with ASD risk [16]. Moreover, several independent genome-wide scans have highlighted loci within the long arm of chromosome 7 as potential candidate genes explaining ASD susceptibility [17,18]. Among the potential loci of interest, the reelin gene *(RELN)* maps to 7q22 and encodes a signaling glycoprotein considered to play a key role in the migration of several neuronal cell types and the development of neural connections. Furthermore, Fatemi et al. [19] showed decreased levels of reelin protein in autistic patients. The role of *RELN* in ASD is thus supported by functional studies, as well as genome scans showing ASD linkage peaks in the region that contains this gene.

Several genetic association studies have been performed to analyze the association between genetic variants within the *RELN* gene and ASD, but the results have been conflicting. Some studies have found significant associations between ASD and longer triplet repeats in the 5′UTR region of this gene [20] or certain SNPs. Specifically, attention has been mostly focused on the polymorphisms rs736707, rs362691, and rs2229864. However, while some authors have found positive associations [21,22], other researchers have published negative findings [23,24]. Given these controversial results, the aim of this study is to analyze the association between ASD and genetic variants within the *RELN* gene by means of a systematic review and meta-analysis.

## 2. Materials and Methods 

### 2.1. Inclusion Criteria

In this review, we included case-control studies that analyzed the relationship between ASD and 3 single nucleotide polymorphisms (SNPs) located within *RELN* (rs736707 in intron 59, rs362691 in exon 22, and rs2229864 in exon 50 of *RELN*). We also analyzed the potential association of ASD with a polymorphic trinucleotide repeat (CGG/GCC) within the 5′UTR region of *RELN*. The included reports had to include patients with ASD as cases alongside a control group comprised of healthy unrelated subjects. ASD had to be defined by the use of accepted diagnostic criteria (according to the Diagnostic and Statistical Manual of Mental Disorders, 4th edition (DSM–IV); Diagnostic and Statistical Manual of Mental Disorders, 5th edition (DSM–5); the International Classification of Diseases–10 (ICD–10); the Autism Diagnostic Interview; the Autism Diagnostic Interview-Revised; or the Autism Diagnostic Observation Schedule).

### 2.2. Bibliographic Search and Data Extraction

Reports published before 3 1July 2020 that fulfilled the inclusion criteria were then identified. For this process, a bibliographic search was undertaken in the following databases: Medline (PubMed), Embase, and Web of Science. The terms used to carry out the search were “autism”, “autistic”, “polymorphism”, “genetic variant”, “reelin”, “reln”, “Polymorphism, Genetic”, and “Autistic Disorder”. There were no language restrictions for this study. The search was complemented by reviewing the references of the included articles. Additional reports were retrieved using the PubMed option "Related Articles" and the Web of Science option “Times cited”.

The search and data extraction were independently carried out by three of the authors (C–AJ, HG–I, and TV–HG). 

Each author imported their search results into a reference manager software (Endnote 6) and removed duplicate references at import. The titles and abstracts were screened independently by the authors (C–AJ, HG–I, and TV–HG) for inclusion and exclusion. Disagreements were resolved by consensus. We recorded the following information: authors’ name(s), year of publication, and country. Moreover, allele and genotype frequencies were extracted or calculated from the raw data.

### 2.3. Statistical Analysis

Our meta-analysis compared the presence of the above-mentioned allelic variants among patients with ASD as cases versus healthy unrelated controls. The odds ratio (OR) and its 95% confidence interval (CI) were estimated for each paper. The pooled results are reported as the OR with a 95% CI and *p*-values using a random effects model [25]. A *p*-value < 0.05 was considered statistically significant. Cochran’s Q–statistic was used to assess heterogeneity: a significant Q–statistic (*p* = 0.10) indicated heterogeneity across studies. The I^2^ statistic was used for estimation of the inconsistency in the meta-analyses (percentage of the observed between-study variability due to heterogeneity rather than chance). The following cut-off points were used: I^2^ = 0–25%, no heterogeneity; I^2^ = 25–50%, moderate heterogeneity; I^2^ = 50–75%, large heterogeneity; and I^2^ = 75–100%, extreme heterogeneity [26,27]. In cases with significant results, a sensitivity analysis was carried out to analyze the effect when excluding individual studies in the results.

This meta-analysis was performed using the computer software packages Review Manager 5.4 and MIX v.1.7. [28,29]. As in other meta-analyses [30], the information available for other studies was assessed; for this reason, ethical approval was not required.

## 3. Results

### 3.1. Study Identification and Selection

**Figure 1:**
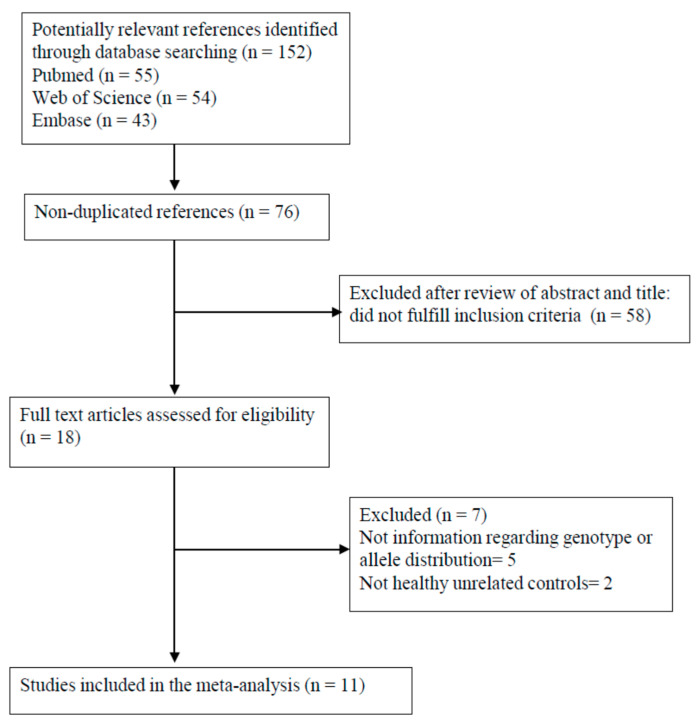
Flowchart of the selection of studies for inclusion in the meta-analysis.

The flow of study identification and selection is shown in Figure 1. Our search strategy detected 152 potentially relevant papers, 18 of which were selected for further analysis. Five of the 18 studies [21,31,32,33,34] were excluded because they did not provide the distribution of SNPs or the distribution of the polymorphic trinucleotide repeat (CGG/GCC) within the *RELN* in the cases and controls.

Moreover, the studies by Krebs et al. [35] and Li et al. [36] were excluded since they only considered cases and parents of cases but not healthy unrelated controls. Therefore, 11 studies [20,22,23,24,37,38,39,40,41,42,43] were ultimately included in our meta-analysis. The allele and genotype distribution among the cases and controls is summarized in Table 1, Table 2 and Table 3. 

Regarding the country of origin, three studies were carried out in China [24,39,43], two studies were performed in India [23,38], two studies were performed in Iran [40,41], and the other studies were performed in Canada [37], South Africa [22], Italy [20], and Turkey [42]. Other demographic characteristics are shown in Table 4.

All studies used genomic DNA extracted from nucleated peripheral blood cells and carried out genotyping using polymerase chain reaction.

### 3.2. Relationship of Reelin Gene Polymorphisms with ASD

**Figure 2:**
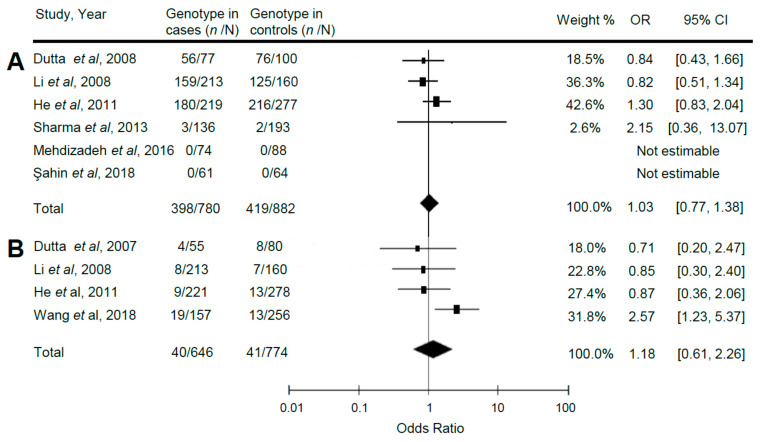

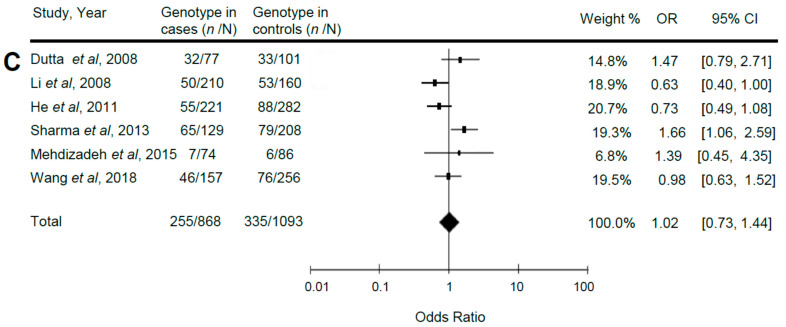
Meta-analysis of the association of reelin gene polymorphisms with autistic spectrum disorder (ASD). (**A)**: Distribution of the genotype CC of the exon 22 polymorphism (rs362691) compared between patients with ASD (cases) and healthy controls under a random effects model. (**B**): Distribution of the genotype TT of the exon 50 polymorphism (rs2229864) compared between patients with ASD (cases) and healthy controls under a random effects model. (**C**): Distribution of the genotype TT of the intron 59 polymorphism (rs736707) compared between patients with ASD (cases) and healthy controls under a random effects model.

The summary and statistics for the association of reelin gene polymorphisms with ASD are shown in Table 5 and Figure 2 (A: Distribution of the genotype CC of the exon 22 polymorphism (rs362691) compared between patients with ASD (cases) and healthy controls under a random effects model. Test for overall effect: Z = 0.21 (*p* = 0.83). Test for heterogeneity: χ^2^ = 2.85 (*p* = 0.42); I^2^ = 0%. B: Distribution of the genotype TT of the exon 50 polymorphism (rs2229864) compared between patients with ASD (cases) and healthy controls under a random effects model. Test for overall effect: Z = 0.49 (*p* = 0.63). Test for heterogeneity: χ^2^ = 5.70 (*p* = 0.13); I^2^ = 47%. C: Distribution of the genotype TT of the intron 59 polymorphism (rs736707) compared between patients with ASD (cases) and healthy controls under a random effects model. Test for overall effect: Z = 0.12 (*p* = 0.90). Test for heterogeneity: χ^2^ = 13.08 (*p* = 0.02); I^2^= 62%). 

We did not find any significant association between possession of the CC genotype of exon 22 (rs362691), TT of exon 50 (rs2229864), or TT of intron 59 (rs736707) and the presence of ASD (Figure 2A: OR = 1.03; 95% CI: 0.77, 1.38; *p* = 0.83; Figure 2B: OR = 1.18; 95% CI: 0.61, 2.26; *p* = 0.63; Figure 2C: OR = 1.02; 95% CI: 0.73, 1.44; *p* = 0.90, respectively). Large heterogeneity was found (I^2^ = 62%, *p* = 0.02) when analyzing the rs736707 polymorphism. The comparison of allele frequencies and other models of inheritance also did not yield any significant results.

### 3.3. Relationship of Polymorphic Trinucleotide Repeat (CGG/GCC) within the Reelin Gene with ASD

**Figure 3:**
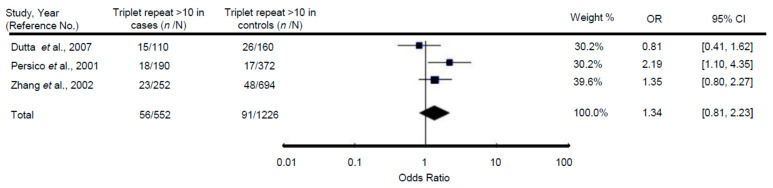
Meta-analysis of the association between the polymorphic trinucleotide repeat (CGG/GCC) within the reelin gene and ASD.

Meta-analysis regarding the association of the presence of >10 repeats of this triplet and ASD did not find any significant association (OR = 1.34; 95% CI: 0.81, 2.23; *p* = 0.26) (Figure 3: The presence of >10 repeats compared between patients with ASD (cases) and healthy controls under a random effects model. Test for the overall effect: Z = 1.13 (*p* = 0.26). Test for heterogeneity: χ^2^ = 3.97 (*p* = 0.14); I^2^ = 50%). The comparison of specific genotype frequencies also did not show any significant relationship (Table 5).

## 4. Discussion

In our meta-analysis, we found no significant relationship between ASD and the distribution of the analyzed SNPs (rs736707, rs362691, and rs2229864) or the presence of longer triplet repeats in the 5′UTR region of the *RELN* gene. It is clear, however, that functional studies [19,44,45] and the results from genome wide scans [17,18] strongly suggest a role for this gene and the reelin protein in the development of ASD.

To explain our negative results, our meta-analysis indicates that previous studies with different ethnic groups found diverse and even contradictory results when analyzing these allelic variants. For instance, Li et al. [39] found that the TT genotype of the rs736707 polymorphism was significantly less prevalent among autistic patients in China compared to the controls, whereas Sharma et al. [22] reported a significantly higher prevalence of the T allele of this SNP among autistic patients of mixed ethnicity from South Africa. This may explain the large heterogeneity found for this polymorphism in the meta-analysis. Further, the G allele of the rs362691 polymorphism has been alternatively identified as the minor [31] or the major allele [22], although similar frequencies have been observed in the same ethnic group. All these results point towards genetic heterogeneity of ASD according to ethnicity, which may be partially responsible for the disparity of the results. Regrettably, an ethnicity analysis was not performed because a low number of studies were finally available for inclusion in this meta-analysis. This was, at least in part, because we were not able to fully combine the results from candidate gene association studies with those from family-based association studies since these studies differed in their design and statistical methods. The inclusion of a small number of studies, and their small sample sizes, are potentially associated with the lack of power to detect a small effect of common variants, and we acknowledge that this may be one of the major shortcomings of our work.

Apart from the risks of false negative results due to ethnicity as a confusion factor and/or low sample sizes, we cannot discount other explanations for our findings. Genetic susceptibility to ASD is probably mediated by many loci, each with a small to moderate effect [46]. Therefore, other genetic factors within this gene may have a relevant role in the development of ASD, such as different polymorphisms or haplotypes within *RELN* [33]. It has been also suggested that a specific paternal 5′UTR-CGG repeat allele effect may be of relevance [38]. Moreover, it is possible that the SNPs of the reelin gene that have been significantly associated with ASD are not directly involved with this disorder but exist in linkage disequilibrium with other functional polymorphisms in a nearby locus. Finally, gene–environment interactions or epigenetic factors may be more important than individual SNPs [47]. All these hypotheses are very difficult to test [48], and we admit that it may be challenging to determine the specific genetic factors associated with ASD, particularly since the relevant genetic bases may be subtle [32,46].

For our study, we selected the four genetic variants which had been more frequently analyzed in previous studies in order to be able to conduct a meta-analysis [20,21,22,23,24,31,32,33,34,35,36,37,38,39,40,41,42,43]. Due to the low sample sizes of previous studies and the low number of studies, it would be of interest to conduct more original gene candidate association studies (in addition to genome-wide association studies, such as the one recently developed by Matoba [16], but, unlike that paper, including healthy unrelated controls) to combine these studies with the meta-analysis. Otherwise, it will remain difficult to detect small or medium effects. Furthermore, the criteria for ASD definitions should be standardized between different studies to decrease heterogeneity.

## 5. Conclusions

In summary, our meta-analysis represents a comprehensive and up-to-date revision of the association of *RELN* genetic variants and ASD, including data from 1289 patients and 1858 controls. In light of the current evidence, no single genetic variant within this gene is clearly associated with the development of ASD. Larger studies in different ethnic groups are needed to establish the role of specific genetic variants within *RELN* in the etiology of this disorder.

## Figures and Tables

**Table 1:** Distribution of *RELN* CGG repeat genotypes.

First Author, Year		N	Number of CGG Repeats																	
			*3/10*	*4/8*	*4/10*	*6/10*	*7/8*	*7/10*		*8/8*	*8/9*	*8/10*	*8/11*	*8/11-15*	*8/12*	*8/13*	*8/14*	*9/10*	*9/13*	*10/10*
(Persico et al., 2001) [20]	1. Patients with ASD ^a^	95		1	1					16		44	0		5	2	0			16
	2. Healthy controls	186		0	0					36		85	3		1	3	1			48
(Zhang et al., 2002) [37]	1. Patients with ASD 2. Healthy controls	126 347	0 1	1 0		0 1	0 1	1 0		16 60	0 2	44 138		8 16				1 1	1 0	40 97
(Dutta el al., 2007) [38]	1. Patients with ASD 2. Healthy controls	55 80								0 1		10 12								31 42
**First Author, Year**		**N**	**Number of CGG Repeats**																	
			***10/11***	***10/11-16***	***10/12***		***10/13***		***10/23***		***12/10***	***12/12***	***12/13***	***13/8***	***13/10***	***13/13***	***14/10***		***15/10***	***16/10***
(Persico et al., 2001) [20]	1. Patients with ASD ^a^	95	0		5		3		1				1							
	2. Healthy controls	186	1		2		6		0				0							
(Zhang et al., 2002) [37]	1. Patients with ASD 2. Healthy controls	126 347		14 28								0 2								
(Dutta el al., 2007) [38]	1. Patients with ASD 2. Healthy controls	55 80									0 3			2 4	11 14	1 1	0 1		0 1	0 1

^a^ ASD: Autistic spectrum disorder.

**Table 2:** Distribution of *RELN* CGG repeat alleles.

First Author, Year		N	Number of CGG Repeats														
			*3*	*4*	*5*	*6*	*7*	*8*	*9*	*10*	*11*	*12*	*13*	*14*	*15*	*16*	*23*
(Persico et al., 2001) [20]	1. Patients with ASD ^a^	95		2				84		86	0	11	6	0			1
	2. Healthy controls	186		0				165		190	4	3	9	1			0
(Zhang et al., 2002) [37]	1. Patients with ASD	126	0	1		0	1	85	2	140	4	5	13	0	0	1	
	2. Healthy controls	347	1	0		1	1	277	3	363	7	12	26	1	2	0	
(Dutta et al., 2007) [38]	1. Patients with ASD	55						12		83		0	15	0	0	0	
	2. Healthy controls	80						18		116		3	20	1	1	1	

^a^ ASD: Autistic spectrum disorder.

**Table 3:** Genotype and allele distribution: intron 59, exon 22, and exon 50.

**First Author, Year**		**N**	***rs736707 (intron 59)***					***rs362691(exon22) L997V***					***rs2229864 (exon 50)***				
			***Genotype distribution***			***Allele distribution***		***Genotype distribution***			***Allele distribution***		***Genotype distribution***			***Allele distribution***	
			CC	CT	TT	C	T	CC	CG	GG	C	G	TT	CT	CC	T	C
(Dutta et al., 2007) [38]	1. Patients with ASD ^a^	55											4	24	27	32	78
	2. Healthy controls	80											8	31	41	47	113
(Dutta et al., 2008) [23]	1. Patients with ASD	77	11	34	32	56	98	56	20	1	132	22					
	2. Healthy controls (exon 22 N= 100)	101	19	49	33	87	115	76	23	1	175	25					
(Li et al., 2008) [39]	1. Patients with ASD (intron 59 N= 210)	213	52	108	50	212	208	159	47	7	365	61	8	76	129	92	334
	2. Healthy controls	160	29	78	53	136	184	125	30	5	280	40	7	53	100	67	253
(He et al., 2011) [24]	1. Patients with ASD (exon 22 N= 219)	221	50	116	55	216	226	180	36	3	396	42	9	73	139	92	350
	2. Healthy controls (exon 22 N= 277) (exon 50 N= 278)	282	48	146	88	242	322	216	53	8	485	69	13	87	178	113	443
(Sharma et al., 2013) [22]	1. Patients with ASD (intron 59 N= 129)	136	14	50	65	78	180	3	16	117	22	250					
	2. Healthy controls (intron 59 N= 208)	193	35	94	79	164	252	2	34	157	38	348					
(Mehdizadeh et al., 2015) [40]	1. Patients with ASD	74	41	26	7	108	40										
	2. Healthy controls	86	52	28	6	132	40										
(Mehdizadeh et al., 2016) [41]	1. Patients with ASD	74						0	16	58	16	132					
	2. Healthy controls	88						0	28	60	28	148					
(Wang et al., 2018) [43]	1. Patients with ASD	157	33	78	46	144	170						19	70	68	108	206
	2. Healthy controls	256	54	126	76	234	278						13	76	167	102	410
(Şahin et al., 2018) [42]	1. Patients with ASD	61						0	10	51	10	112					
	2. Healthy controls	64						0	8	56	8	120					

^a^ ASD: Autistic spectrum disorder.

**Table 4:** Characteristics of the studies included in the meta-analysis.

First Author, Year	Country	Criteria for ASD ^a^ Definition	Ethnicity	Female/Male Ratio		Age (Mean [SD])	
				Patients with ASD	Healthy Controls	Patients with ASD	Healthy Controls
Persico et al., 2001 [20]	Italy	DSM–IV ^b^ criteria for Autistic disorder	Caucasian	6/89	89/97	6.25 (2.8)	51.7 (19.6)
Zhang et al., 2002 [37]	Canada	ADI–R ^c^ algorithm / ADOS ^d^	N/A ^e^	N/A	170/177	N/A	N/A
Dutta et al., 2007 [38]	India	DSM–IV criteria for Autistic disorder	Indian	N/A	N/A	N/A	N/A
Dutta et al., 2008 [23]	India	DSM–IV criteria for Autistic disorder	Indian	13/64	N/A	5.8 (2.9)	N/A
Li et al., 2008 [39]	China	DSM–IV criteria for Autistic disorder or ICD-10 ^f^	Chinese Han	32/181	25/135	5.3 (N/A)	6.7 (N/A)
He et al., 2011 [24]	China	DSM–IV criteria for Autistic disorder	Chinese Han	35/197	43/240	N/A	32.8 (10.5)
Sharma et al., 2013 [22]	South Africa	DSM–IV criteria for Autistic disorder	Black, white, and mixed ancestry	N/A	N/A	N/A	N/A
Mehdizadeh et al., 2015 [40]	Iran	DSM–IV criteria for Autistic disorder	Caucasian	18/53	65/21	8.57 (N/A)	N/A
Mehdizadeh et al., 2016 [41]	Iran	DSM–IV criteria for Autistic disorder	Caucasian	18/53	66/22	8.57 (0.07)	7.79 (0.14)
Wang et al., 2018 [43]	China	DSM–IV criteria for Autistic disorder	Chinese Han	21/108	72/184	8.4 (3.9)	8.3 (3.9)
Şahin et al., 2018 [42]	Turkey	^g^ DSM–5 criteria for Autistic disorder	N/A	5/56	12/52	5.54 (3.1)	6.43 (4.0)

^a^ ASD: Autistic Spectrum Disorder. ^b^ DSM–IV: Diagnostic and Statistical Manual of Mental Disorders, 4th edition. ^c^ ADI–R: Autism Diagnostic Interview-Revised. ^d^ ADOS: Autistic Diagnostic Observation Schedule. ^e^ N/A: not available. ^f^ ICD–10: International Classification of Diseases–10. ^g^ DSM–5: Diagnostic and Statistical Manual of Mental Disorders, 5th edition.

**Table 5:** Comparison of the allele and genotype frequencies of genetic variants in patients with autistic spectrum disorder (cases) versus healthy controls under a random effects model.

Polymorphisms	OR	95% CI	*P*_overall effect_	Q	*P*_heterogeneity_
Exon 22					
C vs. G	0.95	0.76, 1.20	0.68	5.32	0.38
CC vs. CG + GG	1.03	0.77, 1.38	0.83	2.85	0.42
GG vs. CG + CC	1.20	0.83, 1.75	0.34	4.08	0.54
Exon 50					
C vs. T	0.81	0.55, 1.19	0.28	13.56	0.004
CC vs. CT + TT	0.75	0.48, 1.16	0.19	11.50	0.009
TT vs. CT + CC	1.18	0.61, 2.26	0.63	5.70	0.13
Intron 59					
C vs. T	0.98	0.77, 1.24	0.84	16.05	0.007
CC vs. CT + TT	1.02	0.76, 1.37	0.88	7.84	0.17
TT vs. CT + CC	1.02	0.73, 1.44	0.90	13.08	0.02
Triplet repeat number					
4	9.09	1.00, 82.50	0.05	0.01	0.94
8	0.86	0.69, 1.08	0.19	1.30	0.52
10	1.00	0.78, 1.29	0.98	2.77	0.25
11	0.89	0.14, 5.59	0.90	1.63	0.20
12	1.68	0.30, 9.50	0.56	7.41	0.02
13	1.26	0.81, 1.97	0.31	0.22	0.89
14	0.66	0.10, 4.20	0.66	0.08	0.96
15	0.52	0.06, 4.69	0.56	0.00	0.95
16	2.00	0.12, 32.62	0.63	1.52	0.22
